# Enhancing Genome-Scale Model by Integrative Exometabolome and Transcriptome: Unveiling Carbon Assimilation towards Sphingolipid Biosynthetic Capability of *Cordyceps militaris*

**DOI:** 10.3390/jof8080887

**Published:** 2022-08-22

**Authors:** Pattsarun Cheawchanlertfa, Suwalak Chitcharoen, Nachon Raethong, Qing Liu, Pramote Chumnanpuen, Panyawarin Soommat, Yuanda Song, Mattheos Koffas, Kobkul Laoteng, Wanwipa Vongsangnak

**Affiliations:** 1 Department of Zoology, Faculty of Science, Kasetsart University, Bangkok 10900, Thailand; 2 Program in Bioinformatics and Computational Biology, Graduate School, Chulalongkorn University, Bangkok 10330, Thailand; 3 Institute of Nutrition, Mahidol University, Nakhon Pathom 73170, Thailand; 4 Colin Ratledge Center for Microbial Lipids, School of Agriculture Engineering and Food Sciences, Shandong University of Technology, Zibo 255000, China; 5 Omics Center for Agriculture, Bioresources, Food, and Health, Kasetsart University (OmiKU), Bangkok 10900, Thailand; 6 Genetic Engineering and Bioinformatics Program, Graduate School, Kasetsart University, Bangkok 10900, Thailand; 7 Department of Chemical and Biological Engineering, Rensselaer Polytechnic Institute, Troy, NY 12180, USA; 8 Industrial Bioprocess Technology Research Team, Functional Ingredients and Food Innovation Research Group, National Center for Genetic Engineering and Biotechnology BIOTEC, National Science and Technology Development Agency NSTDA, Pathum Thani 12120, Thailand

**Keywords:** *Cordyceps militaris*, genome-scale metabolic model, sphingolipid, metabolic footprinting, metabolic responses, transcriptome

## Abstract

*Cordyceps militaris* is an industrially important fungus, which is often used in Asia as traditional medicine. There has been a published genome-scale metabolic model (GSMM) of *C. militaris* useful for predicting its growth behaviors; however, lipid metabolism, which plays a vital role in cellular functions, remains incomplete in the GSMM of *C. militaris*. A comprehensive study on *C. militaris* was thus performed by enhancing GSMM through integrative analysis of metabolic footprint and transcriptome data. Through the enhanced GSMM of *C. militaris* (called *i*PC1469), it contained 1469 genes, 1904 metabolic reactions and 1229 metabolites. After model evaluation, in silico growth simulation results agreed well with the experimental data of the fungal growths on different carbon sources. Beyond the model-driven integrative data analysis, interestingly, we found key metabolic responses in alteration of lipid metabolism in *C. militaris* upon different carbon sources. The sphingoid bases (e.g., sphinganine, sphingosine, and phytosphingosine) and ceramide were statistically significant accumulated in the xylose culture when compared with other cultures; this study suggests that the sphingolipid biosynthetic capability in *C. militaris* was dependent on the carbon source assimilated for cell growth; this finding provides a comprehensive basis for the sphingolipid biosynthesis in *C. militaris* that can help to further redesign its metabolic control for medicinal and functional food applications.

## 1. Introduction

*Cordyceps militaris* is a medicinal fungus, which has been widely used for the production of commercially important metabolites, such as cordycepin, adenosine, D-mannitol, polysaccharide and ergosterol [1]; these metabolites have beneficial effects on several biological systems, including immune, hematogenic, cardiovascular, respiratory, glandular, and nervous systems. Among these metabolites, cordycepin is recognized as a biologically active metabolite with therapeutic potential, including anti-cancer, anti-tumor, anti-diabetic, anti-obesity, anti-bacterial, anti-fungal, anti-platelet aggregation and anti-inflammatory functions [2,3]. Additionally, cordycepin can be used as a functional ingredient in healthcare products, foods, and cosmetics [4,5]. In natural habitats, the fruiting body of *C. militaris* is commonly found on the decomposing bodies of dead insects under particular conditions [6]. Therefore, the fruiting body of *C. militaris* is rather expensive because its traditional production cannot meet the increasing market demand. Regarding the safety concern of consumers, the product derived from the naturally occurring fruiting body of the parasitized insects may be contaminated with other microorganisms, which leads to the limitation of market expansion of this alternative medicine [7].

Lately, artificial cultivation has been developed for mycelial and fruiting body production of *C. militaris*. Many studies have been devoted to the process improvement of cordycepin production of *C. militaris* [8]. The cordycepin production process was improved by optimizing the culture medium, such as the fermented carbon sources [9,10]. With advances in high-throughput sequencing technology, the genome sequence of *C. militaris* has been published [11] and its transcriptome was elucidated under different carbon sources [12,13,14]. The transcriptome analysis of *Cordyceps* species revealed that the *Cordyceps* growth and cordycepin production were dependent on carbon sources, in which glucose was the optimal carbon source for biomass production, and sucrose was favorable for the cordycepin production. Xylose was also an alternative carbon source for cordycepin production, although it was less favorable for fungal growth than glucose and sucrose [10]. Further study of the transcriptome of *C. militaris* showed that the genes coding for the catalytic enzymes in the glycolysis pathway were upregulated when glucose was used as a carbon source [15]. In addition, the genes involved in adenosine, methionine and cordycepin biosynthesis were upregulated in the sucrose-grown culture [15]. A recent study also showed that a set of significantly upregulated genes in the xylose-grown culture was enriched in the pentose and glucuronate interconversion and cordycepin biosynthesis [10]. Very recently, Lin et al. [16] have reported that the lipid profiles between nature and cultivated *Cordyceps* spp. were significantly different, and these differential lipid profiles (e.g., sphingolipids and fatty acids) potentially served as effective indicators for discriminating the geographical origin of nature *Cordyceps* spp. or distinguishing *Cordyceps* spp. In addition to the structural component of bilayer membranes, sphingolipids and unsaturated fatty acids have been reported in association with pharmacological functions [17,18,19,20]. Therefore, the comprehensive study of metabolic footprinting in *Cordyceps* species under different cultivations is useful for exploring its metabolic regulation underlying the lipid metabolism and other cellular metabolisms under particular conditions.

Genome-scale metabolic model (GSMM) is a computational tool useful for optimizing cultivation conditions of *C. militaris* to enhance growth and cordycepin production. The earlier *i*NR1329 model of *C. militaris* has been published [13]; nonetheless, it has missing metabolic reactions, especially in lipid metabolism.

Exometabolomics, known as metabolic footprinting, is a powerful approach for differentiating physiological traits of organisms of interest. Accordingly, the non-targeted footprint metabolites were thus considered as inputs together with the significantly expressed genes for reconstructing the genome-scale metabolic model of *C. militaris* in this work. Therefore, we aimed to enhance the genome-scale metabolic model of *C. militaris* through metabolic footprint with the integration of transcriptome data of the cultures grown under varied carbon sources. Initially, the metabolic footprinting was implemented using high-performance liquid chromatography coupled with electrospray ionization quadrupole time-of-flight mass spectrometry (LC/ESI-QTOF-MS). A comparative annotation of metabolic footprint data of *C. militaris* across different carbon sources was done through XCMS and METLIN. Incorporating the identified footprint metabolites into the earlier model [13] was performed in order to reconstruct the new version of GSMM, which was then used as a scaffold for integrative transcriptome analysis across different carbon sources; this study serves for the further development of *C. militaris* cultivation process from carbon assimilation towards the production of fungal mass enriched in targeted metabolites of industrial relevance.

## 2. Materials and Methods

### 2.1. Sample Preparation for Dry Weight and Metabolic Footprint Analysis

*C. militaris* strain TBRC6039 was used in this study. The stock culture was maintained as a mycelial suspension in 10% (*v*/*v*) glycerol at −80 °C. For inoculum preparation, 0.5 mL of stock solution was added in a 250-mL Erlenmeyer flask containing 75 mL of yeast extract-peptone-dextrose (YPD) medium, and the culture was grown at 22 °C with shaking at 250 rpm for 7 days under dark conditions. For the mycelial cultivation, 5% (*v*/*v*) *C. militaris* inoculum was transferred into individual sterile 250 mL jars containing 75 mL of defined medium enclosed with a sterile filter cap. One liter of the defined medium consisted of 40 mM (NH_4_)_2_SO_4_ and 20 g of sugar, which was supplemented with trace elements, including 0.5 g MgSO_4_·7H_2_O, 0.5 g K_2_HPO_4_·3H_2_O, 0.5 g KH_2_PO_4_, 0.1 g CaCl_2_ and 0.1 g FeSO_4_·7H_2_O. The static cultivation was performed at 22 °C for 60 days under dark conditions. The fermentable sugars used for individual cultivations were xylose (C5), sucrose (C12), and glucose (C6). Three biological replicates of each culture condition were used for the analysis.

For dry cell weight determination, the mycelial cells were harvested through a filter paper, washed at least three times with distilled water and then dried using a freeze dryer at −110 °C until a constant weight was obtained. The samples of cell-free broths obtained from the cultures grown at the logarithmic phase were subjected to the metabolic footprint analysis. One milliliter of each cultured broth was freeze-dried, and the lyophilized samples were then extracted with 100% methanol (HPLC grade, 1:10, *v*/*v*) using vortex QL-866 (Qilinbeier, Jiangsu Nantong, China) at 3000 rpm for 3 min. After centrifugation at 12,000 rpm for 2 min, the supernatant was used for further analysis.

### 2.2. Liquid Chromatography-Mass Spectrometry (LC-MS) Analysis

To analyze the footprint metabolites in the cell-free broths of *C. militaris* cultures, LC/ESI-QTOF-MS technique was employed using an Agilent 6545 Q/TOF Mass Spectrometer equipped with a dual ESI source (Agilent Technologies, Santa Clara, CA, USA) for MS analysis. Samples (3 µL) were separated on an Agilent ZORBAX RRHD Eclipse Plus C18 (2.1 × 50 mm^2^, 1.8 µm) using a gradient elution program with aqueous acetonitrile (5–95%, *v*/*v*) at a flow rate of 400 µL/min. Positive ion electrospray ionization mass spectra were acquired over a mass range of 50–1000 Da. The data acquisition and processing were performed using Agilent Mass Hunter Qualitative B.07.00 software (Agilent Technologies, Santa Clara, CA, USA), and the following parameters were used to extract the peaks from the raw data; retention times between 0.00 and 33.00 min, and the mass spectra with the *m*/*z* ions between 50 and 1000.

### 2.3. Metabolite Acquisition and Identification towards Differentially Accumulated Metabolites (DAMs) Analysis

Agilent Mass Hunter Qualitative B.07.00 software was used for metabolite data acquisition of total ion chromatograms (TIC). All spectra were exported for further analysis by using interactive XCMS Online, which is publicly available via XCMS (https://xcmsonline.scripps.edu/, accessed on 24 August 2021) software version 3.7.1 (The Scripps Research Institute, San Diego, CA, USA) [21]. XCMS is “R software” with a freeware program used for peak picking, grouping and comparing the findings. A pairwise comparison between the metabolic footprint data obtained from the fungal cultures using two different carbon sources grown at the logarithmic phase was performed. Metabolite identification was performed using the XCMS online that has the capability to search MS/MS matching against MS/MS data in the METLIN database (https://metlin.scripps.edu/, accessed on 24 August 2021) [22], where *m*/*z* tolerance was set at 10 ppm. For the measurement of accuracy and resolution mass spectrometry, the LC-MS/MS-Q-TOF 6545 was calibrated using the Agilent G1969-85000 with tuning mix solution (10 times dilution). The diluted tuning mix solution (3 µL) was then injected, and the mass range was 3200 *m*/*z* in the positive and negative modes were detected afterward. The information on biological functions and pathways of the relevant compounds was acquired by mapping the detected metabolites into the relevant biological pathways retrieved from the Kyoto Encyclopedia of Genes and Genomes (KEGG) database (www.kegg.jp, accessed on 30 October 2021).

Relative quantification of metabolites was based on chromatographic peak areas of the LC-MS analysis. The data derived from the metabolic footprinting were normalized for the principal component analysis (PCA) via the XCMS Online interface [23]. A heat map representing the hierarchical cluster analysis (HCA) was generated by using the Clustergrammer based on the Euclidean distance matrix [24]. The Venn diagram was constructed using SmartDraw version Web-based platform (SmartDraw, LLC, The Woodlands, TX, USA) (SmartDraw is the Ideal Venn Diagram Software; www.smartdraw.com/venn-diagram/venn-diagram-software.htm, accessed on 24 January 2022). Volcano plots of differentially accumulated metabolites (DAMs) were generated using R package version 3.6.1. For statistical data analysis, one-way analysis of variance (ANOVA) followed by Tukey’s test for the post-hoc analysis was used for multiple comparisons via Minitab version 17.0. The significant DAMs were then identified using |fold change| ≥ 1.5 (or |log_2_ fold change| ≥ 0.5) under *p*-value of ≤ 0.05.

### 2.4. Improvement of GSMM of C. militaris by Incorporating Metabolic Footprint and Transcriptome Data

To identify the metabolic responses of *C. militaris* cultures to the carbon sources tested, the earlier transcriptome data of this fungal strain, which was grown in the same conditions, were retrieved from the BioProject accession numbers of PRJNA416937 (BioSample: SAMN07969453 and BioSample: SAMN07969454) [15], and subjected to the integration with metabolic footprint data to further reconstruct the genome-scale model of *C. militaris*. The *i*NR1329 [13] was used as a template for incorporating the metabolic footprint data of the *C. militaris* cultures using varied carbon sources. Initially, the data of newly identified footprint metabolites, including metabolite names, chemical formula, molecular weight and IUPAC International Chemical Identifier (InChI) using the RAVEN toolbox 2.0 [25], PubChem [26], ChEBI [27], and MetaNetX [28] as databases were prepared and introduced into the *i*NR1329 model. Then, the gap-filling of metabolic reactions was performed by MeGaFiller [29] together with the previous metabolic networks of *C. militaris* [30,31], which were also used to improve the gene-protein-reaction (GPR) associations. Regarding the results of metabolic footprint analysis, the metabolic reactions involved in the biosynthetic pathways of lipids, e.g., sphingolipid and its derivatives, particularly sphinganine, sphingosine, phytosphingosine, and ceramide, were incorporated into the GSMM.

The relevant transport reactions were then added throughout the network connectivity. For model evaluation, the prediction results were validated with the experimental data taken from the previous publication [13]. Biomass production was then set as the objective function for maximizing growth prediction with the flux balance analysis (FBA) by varying different carbon sources, including glucose, fructose, sucrose, xylose, or arabinose. The constraint conditions were set as in the previous report [13]; moreover, the exchange fluxes of ammonium, phosphate, sulphate, H_2_O, and H^+^ were unconstrained to provide basic nutrients for cell growth. Lastly, the biosynthetic capabilities of the enhanced model for sphingolipid and its derivatives were tested by FBA in this study. To explore this, the secretion reaction of corresponding products was set as the objective function to perform FBA. During the model simulation for sphingolipid biosynthetic capability, glucose was set as the sole carbon source with an uptake rate of 25 mmol/g DW/h and a growth rate of 1 h^−1^. The predicted production rates under 1 × 10^−10^ or infeasible results were considered as no production; otherwise, the model gained the corresponding biosynthetic capacity. The simulations were carried out in MATLAB (R2020b) (The MathWorks, Inc., Netik, MA, USA) with the RAVEN 2.0 (SysBioChalmers, Gothenburg, Sweden) [25] and the COBRA Toolbox v.3.0 (The COBRA toolbox developers, Leiden, The Netherlands) [32]. The scripts used for enhancing GSMM of *C. militaris* are available at a public GitHub repository (https://github.com/sysbiomics/Cordyceps_militaris-GSMM, accessed on 19 April 2022).

## 3. Results and Discussion

### 3.1. Quantitative Metabolic Footprinting of C. militaris

To quantify footprint metabolites, *C. militaris* strain TBRC6039 was initially cultured under different carbon sources. The metabolic footprint in association with the fungal growths on xylose (C5 culture), sucrose (C12 culture) or glucose (C6 culture) was further investigated by LC/ESI-QTOF-MS analysis. The PCA by XCMS was performed to determine the metabolic footprint profiles among all samples. The results were calculated using the feature intensities across all samples. The LC-MS data matrix of each sample was subjected to multivariate analysis of PCA (Supplementary Figure S1) that showed three biological replicates of each carbon source tended to group; this indicated a high reproducibility of the metabolic footprint data generation. In addition, the scattering patterns of three datasets of the cultures (xylose, sucrose or glucose) in the two-dimensional plot showed significant differences among the footprint metabolites (Supplementary Figure S1); these results suggested that there was a sufficient reproducibility of the materials, which made them potentially enabling for further qualitative and quantitative analysis.

Footprint metabolites identification was facilitated by the METLIN database (metlin.scripps.edu/index.php, accessed on 24 August 2021) [33,34], which provided potential matches when they were available. The MS/MS spectra and relevant metabolite information were determined using the Human Metabolome DataBase (HMDB) [35], LIPID MAPS [36], ChEBI [27], PubChem [26], and KEGG pathway database [37]. Total detected metabolites were identified in each pairwise comparison of the cultures, including xylose versus glucose (C5 vs. C6), sucrose versus glucose (C12 vs. C6), and xylose versus sucrose (C5 vs. C12) cultures. Detailed information about the identified metabolites for each set of the pairwise comparison, including the metabolite name, fold change, log_2_ fold change, *p*-value, retention time and *m*/*z* value, are shown in Supplementary File S1. A total of 798, 688, and 375 metabolites were detected in the cultures; C5 vs. C6, C12 vs. C6, and C5 vs. C12, respectively. Among these, 278, 218, and 102 detected metabolites (C5 vs. C6, C12 vs. C6, and C5 vs. C12, respectively) did not match with any metabolites in the database, and thus were assigned as unknown metabolites (Supplementary File S1).

### 3.2. Identification of DAMs across Pairwise Comparisons of the Cultures using Different Carbon Sources

**Figure 1:**
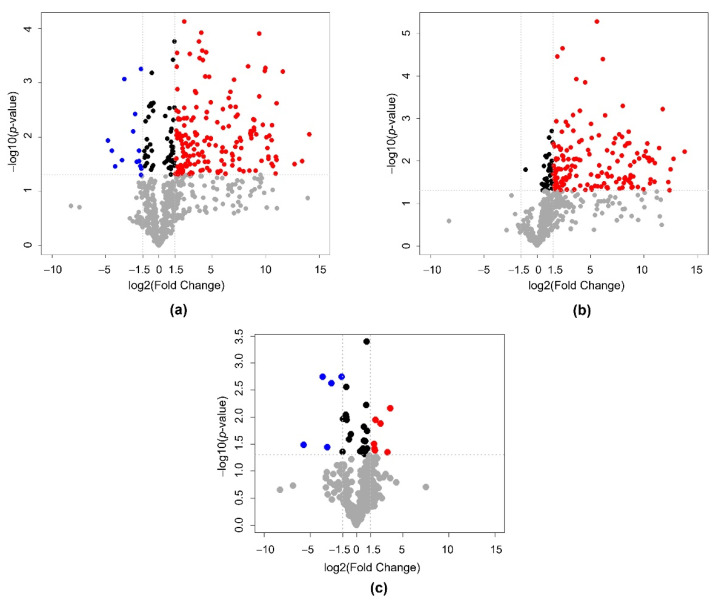
Volcano plots illustrate the significant differences in DAMs between three pairwise comparison sets of the cultures using different carbon sources (**a**) C5 vs. C6 cultures, (**b**) C12 vs. C6 cultures, and (**c**) C5 vs. C12 cultures. Red and blue dots represent the significantly up- and down-accumulated metabolites under |log_2_ fold change| ≥ 0.5 and *p*-value ≤ 0.05, respectively. Gray and black dots indicate DAMs with no significant difference.

To distinguish the footprint metabolites among the cultures using different carbon sources, the DAMs were identified using pairwise comparisons of the metabolic footprint data [38]. The significant DAMs were defined as those exhibiting |log_2_ fold change| ≥ 0.5 and *p*-value ≤ 0.05 between pairwise comparisons of C5 vs. C6 cultures, C12 vs. C6 cultures, and C5 vs. C12 cultures. Detailed information on the DAMs is listed in Table 1 and Supplementary File S2. In total, 252, 188, and 30 significant DAMs were identified in the C5 vs. C6 cultures, C12 vs. C6 cultures, and C5 vs. C12 cultures, respectively. For the C5 vs. C6 cultures, 212 out of 252 metabolites (84.1%) showed increased accumulation (up-accumulation), while 40 out of 252 metabolites (15.9%) showed decreased accumulation (down-accumulation). Of the 188 DAMs identified between the C12 vs. C6 cultures, 187 (99.5%) and 1 (0.5%) metabolites were up- and down-accumulated, respectively. Of the 30 metabolites differentially accumulated in the C5 culture as compared to the C12 culture, 17 (56.7%) and 13 (43.3%) metabolites were up- and down-accumulated, respectively. The DAMs identified in two pairwise comparison sets, C5 vs. C6 cultures and C12 vs. C6 cultures, accounted for 31.6% and 27.3% of all identified metabolites (Supplementary File S1), demonstrating the abundant diversity of metabolites in response to alteration in fluxes of different sugars; moreover, the highest number of DAMs was detected when comparing the C5 vs. C6 cultures, suggesting that the xylose-grown culture exhibited a distinct metabolic profile compared to the glucose-grown culture, respectively. Volcano plots were also generated to represent the significant differences of DAMs between three pairwise comparison sets, C5 vs. C6, C12 vs. C6, and C5 vs. C12 cultures, as shown in Figure 1a–c, respectively.

**Figure 2:**
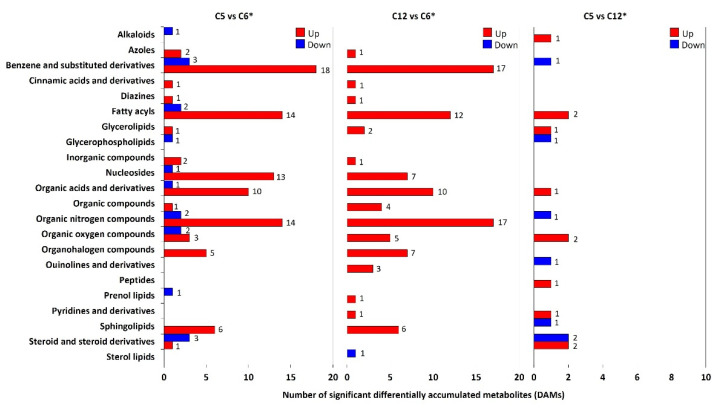
The significant differentially accumulated metabolites (DAMs) across pairwise comparison sets of the cultures using different carbon sources. The bar chart shows the number of significant DAMs in each set of pairwise comparisons. The *x*-axis represents the number of significant DAMs, and the *y*-axis shows the known classes according to the metabolite database (Supplementary File S2). The asterisk (*) means the reference carbon source used for all possible pairwise comparisons.

There were 225 out of 470 significant DAMs in all pairwise comparison sets of the cultures using different carbon sources, which were categorized into 22 known classes (Supplementary File S2). The remaining significant DAMs with unidentified classifications were assigned to the “unknown” class. In the three sets of pairwise comparisons, the significant DAMs were dominantly presented in the organic nitrogen compounds that were benzene and substituted derivatives, fatty acyls, organic acids and derivatives, as well as nucleosides. The highest number of significant DAMs was found in the C5 vs. C6 cultures (109 DAMs with known classes) when compared to the other pairwise comparison sets, suggesting that the xylose culture exhibited a distinct metabolic profile compared to the glucose cultures. In addition, the DAMs belonging to alkaloid, steroid and steroid derivatives were observed in both C5 vs. C6 cultures and C5 vs. C12 cultures, while the sterol lipid was identified specifically in the culture with sucrose as a sole carbon source. To investigate the footprint metabolites characteristics of each sample, the numbers of significant DAMs were grouped into their respective classes, as illustrated in Figure 2. In the comparison of C5 vs. C6 cultures, the DAMs were mostly up-accumulated, whereas a few DAMs in the classes of alkaloid, benzene and substituted derivatives, fatty acyls, glycerophospholipids, nucleosides, organic acids and derivatives, organic nitrogen compounds, organic oxygen compounds, prenol lipids, steroid and steroid derivatives were down-accumulated. For the C12 vs. C6 cultures, almost DAMs were also up-accumulated; nonetheless, the number of DAMs with up- and down-accumulated was comparable in the C5 vs. C12 cultures. Taken together, these results indicated that the comparison sets of C5 vs. C6 cultures and C12 vs. C6 cultures exhibited highly abundant and diverse footprint metabolites composition compared to the pairwise comparison set of C5 vs. C12 cultures; this finding suggests that an alteration of carbon source for fungal cultivation might attribute to the metabolic adaptation in cellular metabolisms and other relevant metabolic processes for mycelial growth.

### 3.3. Identification of Significant Abundant DAMs in the Cultures Using Different Carbon Sources

**Figure 3:**
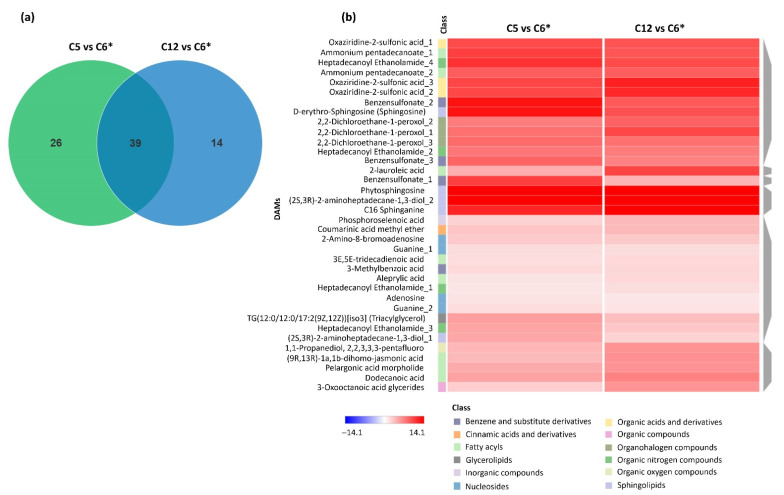
The distribution of significant DAMs in the *C. militaris* cultures as pairwise comparisons between the C5 vs. C6 cultures and C12 vs. C6 cultures (**a**). Venn diagram shows a number of common DAMs across pairwise comparison sets (**b**). A heat map diagram shows patterns of DAMs as pairwise comparisons together with the identified metabolites and relevant compound classes. Each of DAMs is colored by log_2_ fold change value. The asterisk (*) means the reference carbon source used for all possible pairwise comparisons.

To investigate the metabolites commonly and specifically accumulating in response to the type of carbon sources, significant DAMs were identified. As seen in Figure 3, it was found that 39 metabolites accumulated in common among the C5 vs. C6 cultures and the C12 vs. C6 cultures (Supplementary File S3). As clearly observed, the common DAMs were assigned into the 12 known classes (Figure 3a), such as benzene and substituted derivatives, cinnamic acids and derivatives, fatty acyls, glycerolipids, inorganic acid compounds, nucleosides, organic acids and derivatives, organic compounds, organohalogen compounds, organic nitrogen compounds, organic oxygen compounds, and sphingolipids, suggesting that these metabolites might be generated during the fungal growth through either xylose or sucrose assimilation. When considering all possible pairwise comparison sets, interestingly the intermediate metabolites in sphingolipids, including (2S,3R)-2-aminoheptadecane-1,3-diol, sphinganine, sphingosine, phytosphingosine, and ceramide, were the most statistically significant metabolites among three pairwise carbon sets, with high levels in the C5 vs. C6 cultures (i.e., the xylose and glucose cultures); it has been reported that the sphingolipids, which are a fundamental component of the cellular membrane, were detected in the exotic *Cordyceps* [17,39]. In addition, sphinganine is categorized into sphingoid-based metabolites, which is one of the intermediates in the sphingolipid biosynthetic pathway of *C. militaris* (www.genome.jp/kegg, accessed on 30 October 2021). Further, we examined the identified 39 metabolites that were commonly accumulated between the C5 vs. C6 cultures versus the C12 vs. C6 cultures by hierarchical clustering analysis to assess the patterns of significant DAMs between the xylose or sucrose culture when compared to the glucose culture. The heat map result is shown in Figure 3b, indicating that the metabolites assigned into the class of fatty acyls, sphingolipids, nucleosides, and organic nitrogen compounds, were mostly represented.

Promisingly, the metabolites in the sphingolipids class were more greatly accumulated in responses to the C5 vs. C6 cultures, and the C12 vs. C6 cultures, including C16 sphinganine (METLIN: 41556), (2S,3R)-2-aminoheptadecane-1,3-diol_1 and (2S,3R)-2-aminoheptadecane-1,3-diol_2 (METLIN: 479551), D-erythro-sphingosine (sphingosine) (METLIN: 41557), and phytosphingosine (METLIN: 297165). Most of the common significant DAMs were up-accumulated between two pairwise comparison sets, in which 26 and 14 metabolites were specifically accumulated in the C5 vs. C6 cultures and C12 vs. C6 cultures, respectively (Figure 3). Among them, 8 metabolites in fatty acyls, glycerophospholipids, and sphingolipids classes, including methyl jasmonate, N-isobutyl decanamide, decanoic acid, dilauryl tartrate, (R)-(+)-1,1,1-trifluoro-2-octanol, PA (17:0/14:1(9z)) (phosphatidic acid), monoethanolamine myristate, and ceramide (Cer(d18:1/20:0)), were particularly found in the C5 vs. C6 cultures. Observably, 3 metabolites, namely 17,20-dimethyl prostaglandin F1α, ceriporic acid A, and 1-O-hexadecyl-2-desoxy-2-amino-sn-glycerol, were up-accumulated in the C12 vs. C6 cultures. Four significant DAMs, including decanoic acid, dilauryl tartrate, (R)-(+)-1,1,1-trifluoro-2-octanol, and PA (17:0/14:1(9z)) (phosphatidic acid), specifically observed in the C5 vs. C6 cultures were down-accumulated. The results showed that the accumulation of sphingolipid and its derivatives (e.g., sphinganine, phytosphingosine, sphingosine, and ceramide) was substantially affected by carbon sources. Several of these metabolites were relevant to lipids and fatty acids, among which some of them were recognized as potential bioactive compounds, such as phytosphingosine and ceramide; these data further indicated that lipids might play significant roles in the fungal growth under particular conditions, such as the type of carbon sources. Particularly, the sphingolipids might be crucial metabolites in *C*. *militaris* according to their structural and functional roles; these complex lipids constitute a sphingoid base as a backbone in which a fatty acyl and specific head groups are attached, such as dihydrosphingosine (sphinganine), phytosphingosine, or sphingosine; they are found on both the inner and outer membranes of eukaryotic cells [40], and thus are responsible for modulating cell responses as signaling and regulatory molecules. For biological roles, it has been reported that the natural sphingolipid (myriocin) isolated from the culture broth of *Cordyceps* *sinclairii* suppressed the proliferation of lymphocytes in mouse allogeneic mixed lymphocyte reaction [41]; moreover, the phytosphingosine has been reported to be a bioactive molecule with anti-microbial, anti-inflammatory, and anti-cancer functions [42].

Considering the sphingolipid biosynthesis, ceramides are precursors for sphingomyelins (SMs). The formation of ceramide leads to the biosynthesis of a group of metabolites, such as ceramide-1-phosphate (C1P), sphingosine, and sphingosine-1-phosphate (S1P), which are key regulators of inflammation mechanism [43]. Previous work on biologically active sphingolipid biosynthesis, particularly in the ceramide and S1P-mediated pathways, has shown that these molecules have central roles in cancer and type II diabetes [44]. Additionally, a prior work showed that sphingosine can recognize the receptors on conidia, and induce the germination of *Nomuraea rileyi* [45]; this finding might be applied to a biological pesticide, which plays a role in the host recognition mechanism and reduces the time between conidia attachment and their invasion into the insects.

Additionally, the results of pairwise comparisons of both metabolite sets of *C. militaris* cultures using different carbon sources (Supplementary File S3) revealed that the content of adenosine and guanine involved in the purine metabolism significantly increased; it is noteworthy that adenosine is a well-known direct precursor of cordycepin in *C. militaris* [46].

### 3.4. Enhancing Genome-Scale Metabolic Model (GSMM) Using Significant Metabolic Footprint Profiles

**Figure 4:**
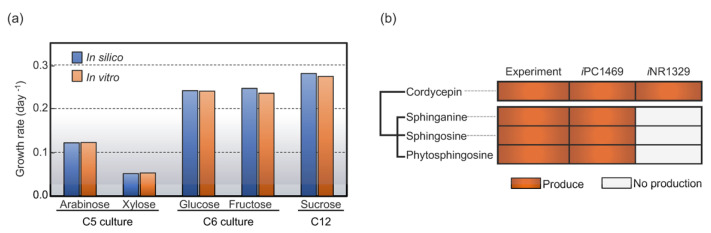
Validation of the enhanced GSMM, *i*PC1469. (**a**) Model validation by comparison of growth rate (day^−1^) between in silico and in vitro data across different carbon sources. (**b**) The predictions of sphingolipid derivatives. Experimental data are shown in the first column, while the modeling results are shown in the rest columns.

To enhance the GSMM of *C. militaris* through the metabolic footprint with integration of transcriptome data of the cultures grown under varied carbon sources. Once incorporating the identified footprint metabolites for reconstructing the new version of GSMM and then used it as a scaffold for integrative transcriptome analysis across different carbon sources. Altogether, the final model *i*PC1469 was developed in this work, which consisted of 1469 genes, 1904 metabolic reactions and 1229 metabolites, as listed in Table 2. Compared with the earlier *i*NR1329 model, the *i*PC1469 included 59 and 83 newly-identified metabolites and metabolic reactions, respectively (see Supplementary File S4 and Supplementary File S5); moreover, 140 unique genes were identified in *i*PC1469. The validation of the enhanced model (*i*PC1469) was performed based on experimental data from the previous study [13]; it was found that the predictive growth rates fitted well with the experimental data with fewer error rates (<5%) upon glucose, fructose, sucrose, xylose, or arabinose, which is consistent to the previous study [13] as shown in Figure 4a; moreover, *i*PC1469 was employed to investigate the biosynthetic capacities of *C. militaris* for sphingolipid derivatives, i.e., sphinganine, phytosphingosine, and sphingosine as shown Figure 4b; this indicated the *i*PC1469 model versatility for sphingolipid and its derivatives production. We suggested that this enhanced GSMM of *C. militaris* could be used as a template model for further cellular metabolism studies of other related fungal species.

### 3.5. Integrative Transcriptome Analysis Using The Enhanced GSMM Revealed Metabolic Response in Lipid Biosynthetic Capability

Using the enhanced GSMM of *C. militaris* (*i*PC1469) together with integrative transcriptomic data, we identified potential metabolites with corresponding differentially expressed genes, which attributed the fungal growths on carbon sources at molecular and biochemical levels. The published transcriptomic data of the *C. militaris*, which was grown in the same culture media and temperature at the dark condition [15] (see Supplementary File S6), was deposited in the BioProject accession numbers of PRJNA416937 (BioSample: SAMN07969453 and BioSample: SAMN07969454), was subjected to integrative data analysis. By comparing results between the xylose vs. glucose cultures as well as the sucrose vs. glucose cultures, we found the up-accumulated metabolite TG; 12:0/12:0/17:2(9Z,12Z) (Triacylglycerol) in the xylose and glucose cultures with log_2_ fold change of 4.35, which was higher than that of the sucrose and glucose cultures (log_2_ fold change of 2.93); this particular metabolite was associated with glycerolipid metabolism. In addition, the differentially accumulated metabolites in the sphingolipid biosynthesis were largely detected when comparing the profiles of the xylose vs. glucose cultures, e.g., sphinganine, phytosphingosine, sphingosine as well as the sucrose vs. glucose cultures, e.g., ceramide. Of them, several key genes involved in the sphingolipid biosynthesis were also specifically activated, corresponding to the altered metabolic footprint profiles. The detailed results are as follows.

#### 3.5.1. Glycerolipid Biosynthetic Pathway

**Figure 5:**
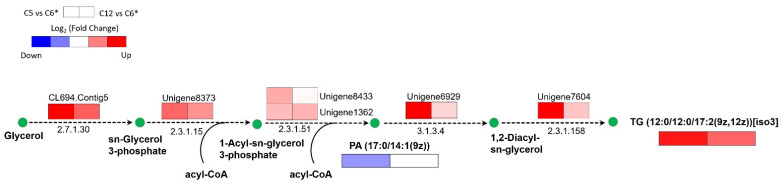
The subnetwork of glycerolipid metabolism in *C. militaris* responsible for the fungal growths on different carbon sources (C5 vs. C6 cultures and C12 vs. C6 cultures) using enhanced GSMM of *C. militaris*. The horizontal boxes under the metabolite name indicate the significant DAMs observed in this pathway, which are PA (17:0/14:1(9z)) and TG (12:0/12:0/17:2(9z,12z)) [iso3] represented as phosphatidic acid and triacylglycerol, respectively. A list of DAMs and DEGs involved in the glycerolipid biosynthetic pathway is listed in Supplementary File S7 (Tables S1 and S2). The asterisk (*) means the reference carbon source used for all possible pairwise comparisons.

Considering the TG; 12:0/12:0/17:2(9Z,12Z) (Triacylglycerol) with up-accumulated content, as expected, a set of upregulated genes was identified in the subnetwork of glycerolipid metabolism through the enhanced GSMM of *C. militaris* (*i*PC1469), including glycerol kinase (EC: 2.7.1.30, CL694.Contig5), glycerol-3-phosphate acyltransferase (EC: 2.3.1.15, Unigene8373), 1-acylglycerol-3-phosphate O-acyltransferase (EC: 2.3.1.51, Unigene8433 and Unigene1362), phosphatidate phosphate (EC: 3.1.3.4, Unigene6929), and phospholipid:diacylglycerol acyltransferase (EC: 2.3.1.158, Unigene7604), which were highly expressed in the xylose vs. glucose cultures as compared with the sucrose vs. glucose cultures (Figure 5). In addition, the PA (17:0/14:1(9z)) (phosphatidic acid), a key intermediate in the glycerolipid biosynthetic pathway, was down-accumulated when the pairwise comparison between the xylose and glucose cultures.

#### 3.5.2. Sphingolipid Biosynthetic Capability

**Figure 6:**
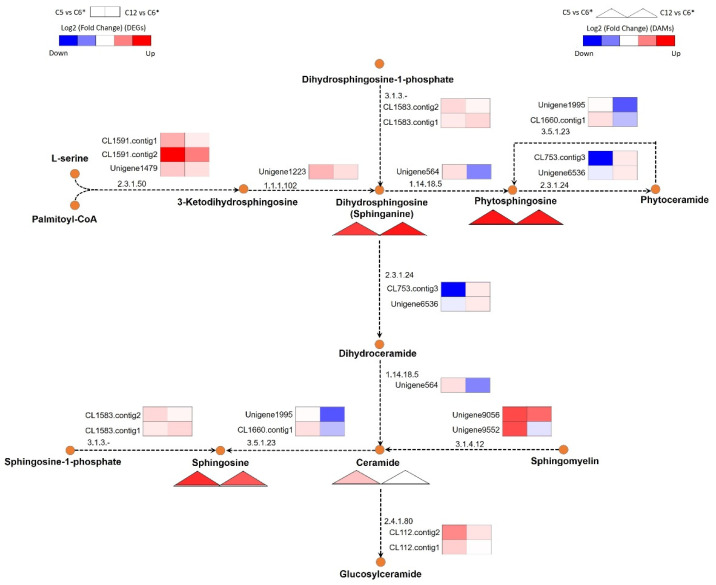
Putative pathway for the sphingolipid biosynthesis in *C. militaris* by integration of the differentially accumulated metabolites (DAMs) and gene expression data of the C5 vs. C6 cultures and the C12 vs. C6 cultures through the enhanced GSMM of *C. militaris* (*i*PC1469). The pathway image is modified from the KEGG database. The triangle shape under the metabolite name indicate the significant DAMs observed in this pathway consisting of sphinganine, sphingosine, phytosphingosine, and ceramide. List of DAMs and DEGs involved in the sphingolipid biosynthetic pathway are listed in Supplementary File S7 (Tables S3 and S4). The asterisk (*) means the reference carbon source used for all possible pairwise comparisons.

The enhanced GSMM of *C. militaris*, *i*PC1469, was further used for the integrative analysis of transcriptome and metabolic footprint data. The sphingolipid biosynthetic pathway in *C. militaris* was elaborated by introducing the genes and their associated metabolites as well as biochemical reactions from the previous study reported by Raethong et al. [15]. To understand the biochemical pathways of *C. militaris* focusing on the sphingolipid biosynthesis under the growths on different carbon sources, we mapped significantly detected metabolites on the relevant pathway, as shown in Figure 6. Based on statistical analysis and subnetwork identification, interestingly, we found key metabolic responses in the lipid metabolism in *C. militaris* upon different carbon sources. Particularly, we found that sphingolipid biosynthetic capability as indicated by sphingoid bases, e.g., sphinganine, sphingosine and phytosphingosine, and ceramide, which were significantly accumulated in the xylose culture (C5 culture) when compared with other cultures throughout enhanced GSMM together with metabolic footprint and transcriptome data of *C. militaris* (Figure 6). De novo sphingolipid biosynthesis started with the condensation of serine and palmitoyl-CoA to produce 3-ketodihydrosphingosine catalyzing by the enzyme serine palmitoyltransferase, then the metabolic pathway continues through a series of enzymatic reactions to yield ceramides, phosphosphingolipids, and glycosphingolipids [47]. In this study, 4 DAMs and 3 DAMs in the sphingolipid biosynthesis were identified in the C5 vs. C6 cultures (the xylose versus glucose cultures), and the C12 vs. C6 cultures (the sucrose versus glucose cultures), respectively (see Supplementary File S7; Tables S3 and S4), of which ceramide was not detected in the pairwise comparison between the sucrose and glucose cultures. Interestingly, we found that the genes involved in the sphingolipid biosynthetic pathway were mainly upregulated between the xylose and glucose cultures, such as Unigene1223 encoding for 3-dehydrosphinganine reductase (EC: 1.1.1.102), Unigene564 encoding for sphinganine C4-monooxygenase (EC: 1.14.18.5), Unigene9056 and Unigene9552 encoding for sphingomyelin phosphodiesterase (EC: 3.1.4.12), and CL1583.contig1 and CL1583.contig2 encoding for sphingosine-1-phosphate phosphatase1 (EC: 3.1.3.-). In contrast, the sphinganine C4-monooxygenase (Unigene564) and sphingomyelin phosphodiesterase (Unigene9552) were down-regulated when comparing the sucrose and glucose cultures with the log_2_ fold change values being 0.13 and 0.03, respectively; this might support the absence of ceramide when comparing between the sucrose and glucose cultures.

## 4. Conclusions

The enhanced GSMM (*i*PC1469) model of *C. militaris* was reconstructed in this study and applied with the integration of exometabolome and transcriptome data. In addition to the biomass production, it unveiled sugar utilization towards the lipid biosynthetic capability upon carbon sources, particularly in the sphingolipid and glycerolipid biosynthesis, which might contribute to cellular functions of the fungal cells; this finding serves to further redesign its metabolic control of *C. militaris* for medicinal and functional food applications.

## Figures and Tables

**Table 1:** The significant differentially accumulated metabolites (DAMs) across three pairwise comparison sets of the cultures using different carbon sources.

Pairwise Comparison Sets	Number of Significant DAMs	Up-Accumulated Metabolites	Down-Accumulated Metabolites
C5 vs. C6 cultures	252	212	40
C12 vs. C6 cultures	188	187	1
C5 vs. C12 cultures	30	17	13

Note: DAMs were considered significant difference under |log_2_ fold change| ≥ 0.5 and *p*-value ≤ 0.05. C5, C6 and C12 represent xylose, glucose and sucrose, respectively.

**Table 2:** Comparative metabolic characteristics of the genome-scale models of *C. militaris*.

Characteristics	*i*NR1329 *	*i*PC1469 (This Study)
Number of Genes	1329	1469
Number of Metabolites	1171	1229
Number of Reactions	1821	1904
Enzymatic reactions	1391	1404
Transport reactions	271	339
Exchange reactions	137	140
Spontaneous reactions	21	21
Biomass synthesis reaction	1	1
Biosynthetic capacities of GSMM	Cordycepin	Cordycepin, sphinganine, phytosphingosine, and sphingosine

* Data were taken from Raethong et al. [13].

## Data Availability

Not applicable.

## Supplementary Materials

The following supporting information can be downloaded at: https://www.mdpi.com/article/10.3390/jof8080887/s1, Figure S1: Principal component analysis (PCA) of the footprint metabolites in the xylose, sucrose and glucose cultures; Supplementary File S1: The identified footprint metabolites derived from each pairwise comparison of the cultures using different carbon sources; Supplementary File S2: Differentially accumulated metabolites (DAMs) in each pairwise comparison of the cultures using different carbon sources; Supplementary File S3: Overview of DAMs commonly or specifically accumulated in each pairwise comparison of the cultures using different carbon sources; Supplementary File S4: List of the newly-identified metabolites in the enhanced GSMM, *i*PC1469; Supplementary File S5: The enhanced genome-scale metabolic model (GSMM, *i*PC1469) of *C. militaris*; Supplementary File S6: Transcriptomic data of *C*. *militaris* TBRC6039; Supplementary File S7: List of DEGs and DAMs involved in the glycerolipid and sphingolipid biosynthetic pathways in the xylose versus glucose cultures, and the sucrose versus glucose cultures.
