# Supplementary material for: Enhancing Genome-Scale Model by Integrative Exometabolome and Transcriptome: Unveiling Carbon Assimilation towards Sphingolipid Biosynthetic Capability of Cordyceps militaris
Source: J Fungi (Basel). 2022 Aug 22;8(8):887. doi: 10.3390/jof8080887 (PMC9409897; doi:10.3390/jof8080887)
Supplement: Supplementary file 1 [file jof-08-00887-s001.zip › Supplementary Figure S1.pdf]

Supplementary Figure S1

Enhancing Genome-Scale Model by Integrative Exometabolome and Transcriptome: Unveiling Carbon Assimilation towards Sphingolipid Biosynthetic Capability of *Cordyceps militaris*

Pattsarun Cheawchanlertfa [1,†], Suwalak Chitcharoen [1,2,†], Nachon Raethong [3], Qing Liu [4], Pramote Chumnanpuen [1,5], Panyawarin Soommat [1,6], Yuanda Song [1,4], Mattheos Koffas [7], Kobkul Laoteng [8,*], Wanwipa Vongsangnak [1,5,*]

1 Department of Zoology, Faculty of Science, Kasetsart University, Bangkok 10900, Thailand
2 Program in Bioinformatics and Computational Biology, Graduate School, Chulalongkorn University, Bangkok 10330, Thailand
3 Institute of Nutrition, Mahidol University, Nakhon Pathom 73170, Thailand
4 Colin Ratledge Center for Microbial Lipids, School of Agriculture Engineering and Food Sciences, Shandong University of Technology, Zibo 255000, China
5 Omics Center for Agriculture, Bioresources, Food, and Health, Kasetsart University (OmiKU), Bangkok 10900, Thailand
6 Genetic Engineering and Bioinformatics Program, Graduate School, Kasetsart University, Bangkok 10900, Thailand
7 Department of Chemical and Biological Engineering, Rensselaer Polytechnic Institute, Troy, NY 12180, USA
8 Industrial Bioprocess Technology Research Team, Functional Ingredients and Food Innovation Research Group, National Center for Genetic Engineering and Biotechnology BIOTEC, National Science and Technology Development Agency NSTDA, Pathum Thani 12120, Thailand
* Correspondence: kobkul@biotec.or.th (K.L.); wanwipa.v@ku.ac.th (W.V.)
† These authors contributed equally to this paper.

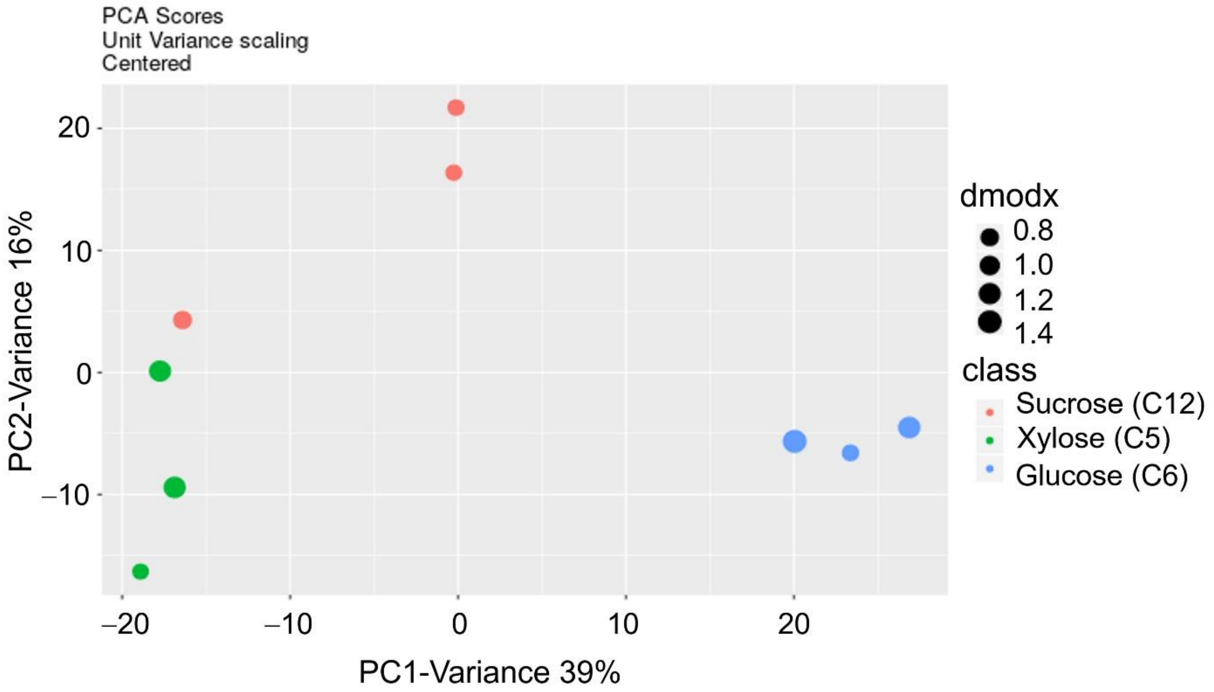

Figure S1. Principal component analysis (PCA) of the footprint metabolites in the xylose, sucrose and glucose cultures. PC1 and PC2 refer to the first and second principal components, respectively.
